# Distributed team processes in healthcare services: a scoping review

**DOI:** 10.3389/fmed.2023.1291877

**Published:** 2023-12-13

**Authors:** Jarle Eid, Guttorm Brattebø, Johan K. Jacobsen, Roar Espevik, Bjørn Helge Johnsen

**Affiliations:** ^1^Centre for Crisis Psychology, University of Bergen, Bergen, Norway; ^2^Department of Anaesthesia and Intensive Care, Norwegian National Advisory Unit on Emergency Medical Communication, Haukeland University Hospital, Bergen, Norway; ^3^Department of Clinical Medicine, University of Bergen, Bergen, Norway; ^4^Safetec Nordic AS, Oslo, Norway; ^5^Department of Leadership, Command and Control, Swedish Defence University, Stockholm, Sweden; ^6^Department of Psychosocial Science, University of Bergen, Bergen, Norway

**Keywords:** patient safety, healthcare, distributed teamwork, coordination, shared mental model, prehospital

## Abstract

**Objective:**

High-quality healthcare services is delivered by teams rather than individuals and depends heavily on multidisciplinary cooperation between dispersed healthcare professionals. The aim of this scoping review is to identify common barriers and innovative applications of technology supporting team processes and patient safety, in geographically dispersed healthcare services.

**Methods:**

Studies were identified from searches in APA PsychINFO, Epistemonikos and Medline databases, from 2010 to 2023. A detailed search strategy was performed, and studies were included, based on prior established criteria.

**Results:**

Among the 19 studies that fulfilled our inclusion criteria, the majority (85%) were from Europe or North America, and most studies (53%) were quantitative, with a cross-sectional study design. Several reported observed distributed team processes in training and education. Most studies described barriers and detailed how innovative approaches and technological solutions were introduced to improve communication, coordination, and shared mental models in distributed healthcare settings. A small proportion of studies (16%) used health services data to examine interpersonal exchange and team processes.

**Conclusion:**

The scoping review offer recommendations to enhance future research on distributed team processes in healthcare services.

## Introduction

Modern healthcare depends on teamwork and cooperation between healthcare professionals (1, 2). However, accumulating evidence suggests that human factors and psychological processes may compromise patient care due to staff distress and communication issues (3–5). The significance of team composition, team processes, assessment and training of healthcare teams are seen as key factors in understanding how non-technical skills influence patient safety (6, 7).

Most research on team processes and team training in healthcare have focused on specific teams, such as trauma teams, or hospital units, like anesthesia and surgery, where critical decisions and effective patient care depend on the physical presence of, and direct coordination between subject matter experts (4, 8, 9). The rapid development of information communication technologies and an increased demand for high-quality prehospital services, have spurred a need for improving the coordination and training of geographically distributed healthcare providers. The COVID19 pandemic prompted a surge in the application of remote technology to enhance the simulation, training, and coordination of geographically distributed health personnel. To date, few studies have mapped this literature to identify common barriers and innovative applications of technology in support of team processes, patient care, and safety in distributed healthcare services. This scoping review contributes to filling this gap.

A systematic review and meta-analysis indicate that teamwork is positively related to performance in healthcare teams (10). An influential strand of widely applied research on team processes has been referred to as ‘The big five of teamwork’ (11). According to Salas et al., the five core elements in teamwork are leadership, adaptability, mutual performance monitoring, backup behavior and team orientation (11). The five team processes are closely linked to performance by three coordinating mechanisms: Mutual trust, shared mental models (SMM), and closed-loop communication. These coordinating mechanisms contribute to ensuring that all critical information is relayed to all team members. Trust is seen as key in situations when team members expect potential harm or adversities if fellow team members fail in fulfilling their duties and responsibilities. Trust is a valuable team asset, since it reduces the need for constant performance monitoring, and facilitates team interaction, backup, or support behavior (12). Shared cognitive constructs, and information about system status and function, allow one to make decisions and predict possible outcomes in familiar situations (13). Over time, shared models are gradually developed and maintained through mutual experiences, training or simulations mimicking realistic operational situations and intra team communication. Inherent knowledge about individual and interpersonal knowledge, capabilities and team processes will increase efficiency by reducing the need for explicit coordination (14). Accordingly, shared mental models are more easily established in co-located than in distributed teams, where visual cues and interpersonal interaction are limited or absent (15). Closed-loop communication is an important coordinating mechanism to avoid misunderstandings; and has long been used in aviation and was later adopted by medicine (16–18). Emerging empirical evidence suggests that closed-loop communication has a direct positive effect by enhancing distributed team processes (19). It has been argued that relational communication is important to create emergent affective states like trust and cohesion, while task-oriented communication contribute significant in the creation of accurate mental models (19).

Lack of face-to-face interaction and communication across technical platforms produce barriers of a physical, temporal, perceptual or emotional nature that influence team processes (20). Such barriers could have adverse effects on team leadership, making it more difficult to engage in mutual performance monitoring and thereby foreseeing the need for backup behavior. According to Morrison-Smith and Ruiz, team challenges can often be traced back to tasks, team composition (roles and responsibilities), and distribution of workload (21). Virtual teams are rife with complex challenges, making such distributed teams less effective than face-to-face teams (22). Reduced efficiency may, in turn, lead to an increased risk of relocation and rotation of the team members, which could reduce cohesion, social relations and team orientation (20, 23). Several studies have shown that familiar teams outperform novel teams with new members in high-fidelity operational situations, such as military or police operations (24–26). Studies on the latter have shown that familiar teams increased their performance in both technical and non-technical (i.e., interpersonal) skills, compared to unfamiliar teams. This relationship between familiar teams and performance was mediated by superior team coordination (26). In a meta-analytic study Mesmer-Magnus et al., concluded that distributed teams, compared to face-to-face teams, needed longer time to fulfill task and showed increased frequency of task-oriented communication contrasted to team oriented communication (47). Furthermore, the inherent challenges in the use of technological platforms for communication between team members also increase the need for more studies on virtual teams. Marlow et al. reported that a common finding regarding communication in distributed teams is a loss of richness in the information transfer (48). Subsequently, the impact of virtuality on the mechanisms between communication and performance as well as the simultaneous moderating effect of contextual factors on this relationship are still not fully examined (48).

While research has shown that non-technical skills, trust, effective communication, virtuality and shared mental models, all are important factors for avoiding mistakes and ensuring safe procedures and reliable performance in co-located healthcare teams. Less research has focused on geographically distributed healthcare teams (1, 9). In this scoping review, we therefore aimed to explore the following four research questions: (1) What barriers will prevent effective healthcare services in geographically dispersed teams? (2) How can technology enhance training, patient safety, and quality of care in distributed healthcare services? (3) Will team processes and coordinating mechanisms observed in co-located teams apply to geographically dispersed healthcare services? (4) How could this scoping review inform future research on healthcare services and patient safety?

## Methods

The review was informed by Arksey and O’Malley’s five-stage framework, which alludes to a rigorous process of transparency, enabling replication of the search strategy and study findings (27). The five stages of this framework informed the research process: (1) identifying the initial research questions, (2) identifying relevant studies, (3) study selection, (4) charting the data, and (5) collating, summarizing, and reporting the results.

### Identifying the initial research questions

The primary aim of our review was to provide an overview of empirical research on common barriers and innovative applications of technology, supporting team processes and coordination of geographically dispersed healthcare services, as indicated by the previous research questions. To this end, multiple databases were consulted to build a coherent search strategy and identify relevant empirical research that could inform our research questions.

### Identifying and selecting relevant studies

For the selection of databases, Epistemonikos was chosen due to its focus on evidence-based research in healthcare and technology. Consequently, Ovid Medline was applied from its position as a predominant database for scientific literature in medicine. Lastly, PsychINFO widely considered to be one of the best databases for accessing psychological literature, was selected to capture team and performance-related studies within the healthcare domain. Since we only wanted to include peer review studies, Google Scholar was not used since this database also contain studies that are not peer reviewed. PubMed is a user-friendly interface to search Medline, but in this study, Ovid Medline was used since it allows a more focused search strategy. A wide range of key words related to virtual teamwork, distributed team processes, healthcare and patient safety were initially adopted as search terms to glean a ‘broad coverage’ of the available literature. The search techniques employed health-related subject headings and Boolean operators to narrow and combine the searches. The resulting terms and their Boolean relationships were combined to form ‘Team* AND (patient safety) AND (leadership OR communication) AND (virtual OR distributed)’ as the search strategy for each database (Table 1).

**Table 1:** Key search terms and Boolean operators in the final search term.

‘team*’ AND ‘*patient safety*’ AND ‘*leadership*’ OR ‘*communication*’ AND ‘*virtual*’ OR ‘*distributed’*

Only peer-reviewed empirical studies in English, published from January 2010 to February 2023, in which the words *communication* or *teamwork* were mentioned in the title or abstract were included. Review studies, case reports and opinion papers were excluded. Studies not available in full text or studies focusing on training, quality improvement, teamwork, or team training of co-located units in hospitals were also excluded (Table 2).

**Table 2:** Overview of inclusion and exclusion criteria used in both searches.

Criterion	Inclusion	Exclusion
Time period	2010 to date of search (01.02.2023)	
Language	English	Non-English studies
Type of article	Original research, published in a peer review journals	Articles that were not peer reviewed or original research
Ethics clearance	Studies with approved ethics notification	Ethics notification not reported
Study focus	Teamwork, Health Care, Virtual/ Distributed teams	Studies without a primary focus on health care, medicine, and distributed teamwork
Literature focus	Studies addressing prehospital services, home care, telehealth or virtual/web-based services	Articles that made a passing or token reference to prehospital services. Review articles, editorials, or opinion papers
Population and sample	Multidisciplinary	Studies on samples other than health care workers
Abstract	Articles where the word *communication* was included in the abstract	Articles lacking the word *communication* in the abstract
Open access	Articles that were available in full text or as open access	Articles in journals not available as open access or through the library services

**Figure 1:**
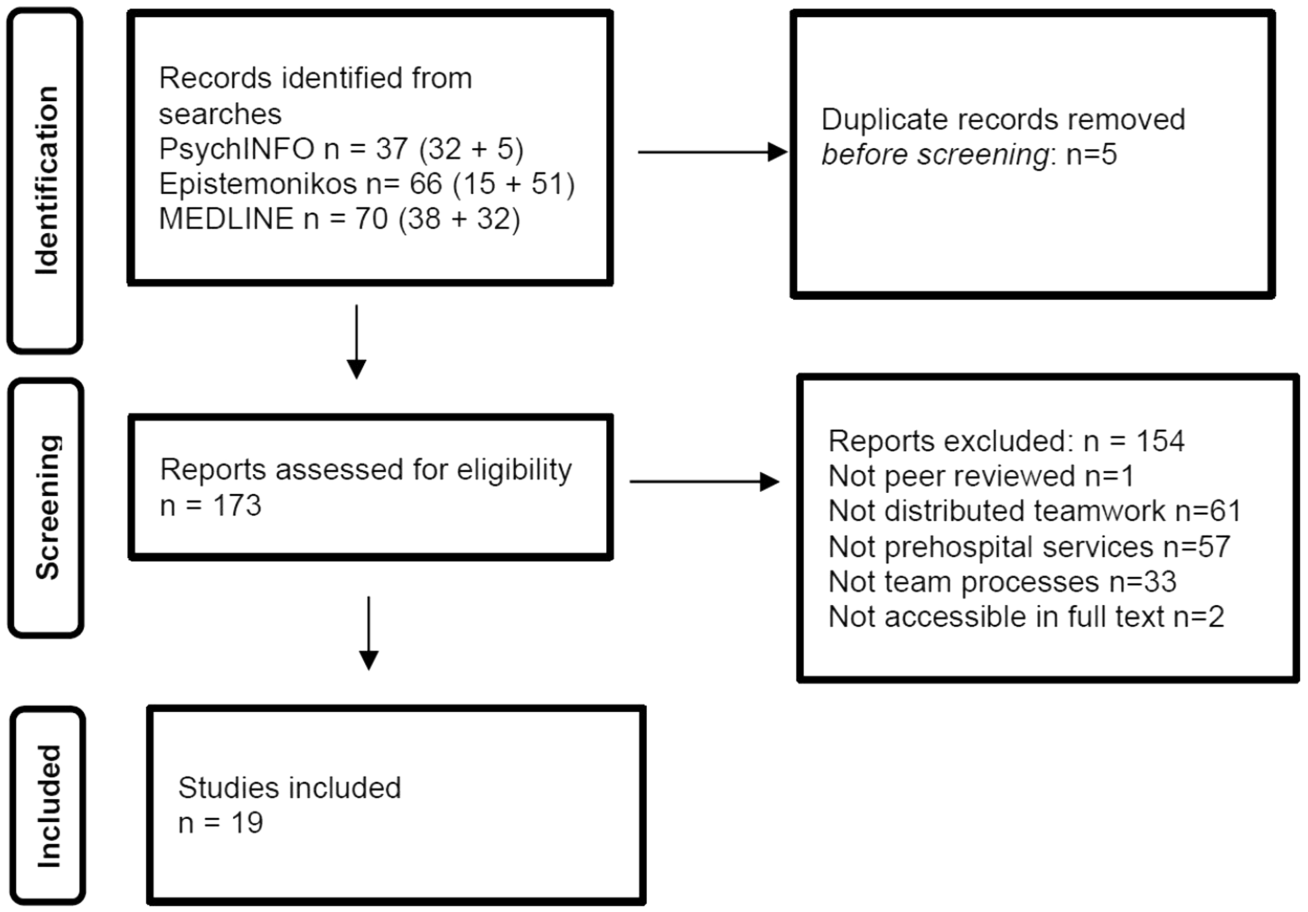
Study flow. Details the flow of information through the different phases of the review; maps out the number of records identified, included and excluded, and the reasons for their exclusion..

A primary database search (from 2010 to 2021) was completed in April 2021 and yielded 85 hits, with 32, 15 and 38 hits from APA PsychINFO, Epistemonikos and Medline, respectively. After the first searches were completed, the researchers conducted a selection process using the Rayyan research review software^1^ to examine the publications and weed out less relevant results (28). After the removal of duplicates and the screening of titles and abstracts, 77 studies were eliminated, and eight studies were retained. To capture relevant research from the COVID-19 period, a supplementary search was completed in the same three databases for the period from April 2021 to February 2023. This search produced 88 additional hits. The first and third author screened the additional studies, using the same exclusion criteria. Finally, 19 studies from the first and the supplementary searches were deemed to fulfill the inclusion criteria and included (Figure 1).

### Data charting, summarizing, and reporting

In the results section summaries are developed for each article related to the authors, publication year, country, study design, data collection, sample size, and a brief descriptive note. The included papers are then narratively summarized with an emphasis on main findings and general domains, followed by a general discussion and recommendation for further research.

## Results

The general characteristics of 19 studies are shown in Table 3. Six studies were conducted in Europe (17, 24–28), one in Asia (34), ten in North America (35–44), and two in Australia (40, 41). Seven studies were quantitative (17, 25–27, 32, 35, 42), three applied mixed methods, and nine applied a descriptive exploratory case study design. The study designs were cross-sectional or descriptive case study designs. No studies applied a longitudinal or a randomized controlled design. Regarding the data collection, five quantitative studies collected data using face-to-face questionnaires, and five studies used databases or online registries. The qualitative studies relied on interviews, video observations, or personal records and observations. The number of subjects in the quantitative studies ranged from 200 to 675 individuals.

**Table 3:** Alphabetic overview of the included studies.

Author details	Title	Location	Study design/participants & sample	Main outcome
Anderson, N., et al. (2020)	Planning for a pandemic: Mitigating risk to radiation therapy service delivery in the COVID-10 era	Melbourne, Australia	Case study: Quality assessment of medical service providers of radiation therapy across campuses and hospitals during COVID-19	Four critical areas were identified in developing risk mitigation strategies across delivery of radiation therapy: (a) Workforce planning, (b) Workforce communication, (c) Patient safety and wellbeing, and (d) Staff safety and wellbeing.
Akşin, Z., Deo, S., Jónasson, J. O., & Ramdas, K. (2021)	Learning from many: Partner exposure and team familiarity in fluid teams.	Turkey	A database study investigated the impact of prior partner exposure on time spent during patient pick-up at the scene and patient handover at the hospital.	For the less standardized patient pick-up process, greater partner exposure directly improved performance. For the more standardized patient handover process, this beneficial effect was triggered beyond a threshold of sufficient individual experience. In addition, the beneficial performance impact from prior partner exposure was amplified in high workload periods.
Bavare A. C. et al. (2021)	Virtual Communication Embedded Bedside ICU Rounds: A Hybrid Rounds Practice Adapted to the Coronavirus Pandemic	Switzerland	Clinical case study: A continuous quality improvement study: Hybrid rounds with virtual communication were introduced during COVID-19 to facilitate social distancing while maintaining patient-centered care.	Hybrid rounds employed during pandemic facilitated social distancing while retaining patient-centered multidisciplinary ICU rounds but compromised teaching during rounds. A change to ingrained rounding habits needs team commitment and ongoing optimization. The hybrid rounds model has potential for generalizability to other healthcare settings.
Dhala, A., et al. (2021)	A Year of Critical Care: The Changing Face of the ICU During COVID-19.	Texas, USA	A case study report on how a tele– critical-care program and its infrastructure were deployed to meet the demands of the pandemic. Community hospitals played a vital role in creating a collaborative ecosystem for the treatment and referral of critically ill patients.	Tele-critical care platforms provided remote monitoring and treatment of ICU patients while extending access to critical care physicians and registered nurses along with decision-support tools necessary for ICU care. A virtual ICU or vICU program was implemented.
Heginbotham, L., et al. (2022)	A parent-led, patient-centered medical home model instruction for interprofessional undergraduate and graduate learning opportunities.	West Virginia, USA	A case study of an educational model to patient-centered medical home (PCMH) to ensure that children with special health care needs are receiving care according to their needs.	The study describes a PCMH training approach that included parents, faculty, and learners in a series of activities (online and in-person) that improve learner knowledge of the PCMH and skills necessary for establishing a PCMH in their future practice.
Hughes, A. M., et al., (2021)	Trauma, teams, and telemedicine: evaluating telemedicine and teamwork in a mass casualty simulation	Chicago, USA	The study examines the effect of telemedical support in a simulated MASCAL simulated training event. Teamwork-related attitudes, behaviors, and cognitions during the MASCAL scenario were measured by pre-post surveys and observations of use.	Overall, clinicians have positive reactions toward the potential benefits of telemedicine; further, participants report a significant decrease in psychological safety after training, with users rating psychological safety as significantly higher than non-telemedicine users.
Hunter, K., et al. (2021)	Feasibility of Prehospital Emergency Anesthesia in the Cabin of an AW169 Helicopter Wearing Personal Protective Equipment During Coronavirus Disease 2019.	UK	Efficiency and outcomes were assessed in a simulated exercise where trained personnel wearing personal protective equipment (PPE) performed a prehospital emergency anesthesia in the form of rapid sequence intubation (RSI).	An in-aircraft RSI (aircraft on the ground) while wearing PPE for AGPs had no significant impact on the time to successful completion of emergency anesthesia (RSI) in a simulated setting.
Husain, A., et al. (2021)	A clinical communication tool (loop) for team-based care in pediatric and adult care settings: hybrid mixed methods implementation study	Canada	The objective of this study was to implement and evaluate the Loop – a web-based, asynchronous clinical communication system for team-based care.	Fundamental structural and implementation challenges persist toward realizing Loop’s potential as a shared system of asynchronous communication. Barriers include health information system integration; system, organizational, and individual tension for change; and a fee structure for health care provider compensation for asynchronous communication.
Johnsen, B. H., et al. (2022)	The Effect of Complexity of Ambulance Missions on Shared Mental Models in Virtual Teams.	Norway	A database study from real life events aimed at mapping team behavior and cognition in critical real-life emergency medical missions based on the concept of SMM.	Voice recordings from real-life missions were used to investigate differences in team behavior between low and high-complexity missions. Lower frequencies of team competencies and coordinating mechanisms were found in high compared to low-complexity missions.
Johnsen, B. H., et al. (2022)	Coordinating mechanisms are more important than team processes for geographically dispersed emergency dispatch and paramedic teams	Norway	A database study investigating the suitability of the Shared Mental Model approach for teamwork between operators in emergency medical communication centers and first line ambulance personnel	Path analyses showed that SMM was positively associated with team effectiveness and negatively related to mission complexity. The coordinating mechanisms of SMM and closed loop communication was positively related to “Big Five” team scores.
Keiser, M. M., Turkelson, C., Smith, L. M., & Yorke, A. M. (2022)	Using Interprofessional Simulation with Telehealth to Enhance Teamwork and Communication in Home Care.	Michigan, USA	A mixed method, observational research design was used to evaluate teamwork and communication following virtual/web-based deliberate practice and a subsequent face-to-face simulation-based interprofessional education activities (Sim-IPE) with a home-based patient assessment and intervention for students in undergraduate nursing, nurse practitioner, and physical therapy programs.	Teams scored very high on an interprofessional communication and teamwork scale, and students strongly agreed that the pre-briefing, scenario, and debriefing assisted in their learning. Students also valued exposure to telehealth and the ability to work with students from other health professions.
Lama, A., Hogg, J., & Olson, A. P. (2020)	Perspectives from the other side of the screen: how clinicians and radiologists communicate about diagnostic errors	Minneapolis, USA	Cross sectional survey: 240 radiologists and clinicians completed a survey on communication and diagnostic errors in health care.	Clinicians and radiologists discover diagnostic errors surrounding the interpretation of radiology images, although radiologists discover them more frequently. There is significant room for improvement in education and practice regarding how radiologists and clinicians communicate as a team.
Miller, W., et al. (2020)	Homecare safety virtual Quality improvement collaboratives	Canada	Descriptive case study: The Canadian Patient Safety Institute and the Canadian Home Care Association conducted two learning collaboratives aimed at increasing quality improvement capability and patient safety practices in homecare settings.	The program engaged teams from across the country to increase their capacity and capability to engage patients and families, mitigate and prevent harm from homecare safety incidents such as falls and specifically address issues such as improving interprofessional collaboration, teamwork, and communication.
Mill, T., et al. (2021)	Live streaming ward rounds using wearable technology to teach medical students: a pilot study.	UK	A pilot study was conducted during COVID-19 exploring the feasibility of using a wearable headset to live stream teaching ward rounds to remotely based medical students. Three live streamed teaching ward rounds were delivered to three groups of medical students using the Microsoft HoloLens 2 device and Microsoft Teams software.	The experience of live streamed ward rounds was well received by patients, medical students, and teaching faculty. However, there remain limitations to the routine use of HoloLens 2 technology including steep learning curves, hardware costs and environmental factors such as noise and WiFi connectivity.
Peddle, M. (2019)	Participant perceptions of virtual simulation to develop non-technical skills in health professionals	Australia	A descriptive exploratory design was used to study responses from 675 health care providers engaged in a virtual simulation program. Most respondents were nurses (81%), with remaining sample from other health professions.	Results indicated that virtual simulation increased awareness of non-technical skills including communication, teamwork, decision making, critical thinking and problem solving, as well as situational awareness.
Reece, S., et al., (2021).	Use of virtually facilitated simulation to improve COVID-19 preparedness in rural and remote Canada	Canada	A feasibility study of an *in situ* virtually facilitated simulations (VFS) for COVID-19 airway management and health systems preparedness that was administered to 200 health care providers in rural Canada.	Video analysis of sequential VFS rapid cycle sessions using a standardized observational tool indicated decreased personal protective equipment (PPE) breaches by 36.6% between the first and third cycles. Teams demonstrated increased competency with airway management and VFS provided a rapidly mobilizable and cost-effective way of delivering high-quality SBE to geographically isolated communities.
Sasangohar, F., et al. (2020)	Adapting an outpatient psychiatric clinic to telehealth during COVID-19: A practice perspective	Houston, USA	Case study: A descriptive report on a rapid transition to a 100% digital outpatient mental health service.	Describes the logistics of the implementation, including modes of communication, the psychological effects of web-based services, including both the loss of the physical therapeutic environment and the unique interpersonal dynamics experienced in the virtual environment.
Umoren, R. A., et al. (2017)	TeamSTEPPS Virtual Teams: Interactive Viertual Team Training and Practive for Health Professional learners	Seattle, USA	Descriptive case study: In 2016, 1,128 unique users accessed Interactive virtual simulation scenarios designed to permit flexible, asynchronous learning and team training	Interprofessional faculty from multiple institutions and specialties created a series of eight screen-based interactive virtual simulation cases featuring typical clinical situations, with the goal of preparing learners to provide safe and effective care in clinical teams.
Wooldridge, A. R., et al., (2019)	Complexity of the pediatric trauma care process: Implications for multi-level awareness	UK	A mixed method design with interview, archival document and trauma registry data were used to describe how intra-hospital care transitions affect process and team complexity.	Identified 53 roles, 4 physical locations and 69 pathways of pediatric trauma care. Process modeling or simulation is suggested to present a potential solution to the complex, distributed nature of the process of trauma care and the roles and interdependencies within the process.

Taken together, six studies addressed innovative approaches to team training and development (31, 34, 36, 37, 39, 41) ten studies addressed the implementation of new technology or assessed organizational procedures in support of improved healthcare services (24, 25, 27, 28, 30, 32, 33, 35, 38, 40), and three studies utilized registries or database records to identify basic mechanisms in distributed team processes (17, 26, 42). In the following we will chart and collate these findings in more detail.

### Technological innovation in support of team training and education

Several studies detailed team training and the feasibility of technology in support of distributed healthcare practices. Two studies focused on virtual team training (44, 46). In the Team STEPPS program, eight screen-based interactive virtual simulation cases featured typical clinical situations and formed the core of the program (44). In a similar study, virtual simulations were found to be an efficient strategy to facilitate awareness of non-technical skills, communication, and critical thinking (46). By analyzing participant perceptions, these simulations were shown to improve awareness of communication, teamwork, decision making, and problem solving (46). A more general improvement of overall situational awareness was also discovered. Whilst virtual simulations facilitated flexible, asynchronous learning adapted to the student’s schedule, it was challenging for the educators to monitor and provide timely individual feedback.

Four of the training studies were designed and implemented during the COVID-19 pandemic, exploring how virtual reality was introduced in support of distributed healthcare and education. The study by Reece et al. was directly aimed at using virtually facilitated simulation to improve COVID-19 preparedness in 200 healthcare providers in rural Canada (42). Their feasibility study focused on airway management and health systems preparedness as priority objectives. Video analysis and observations indicated that the healthcare teams demonstrated increased competency, as well as cost-effectiveness and feasibility of virtual training to reach geographically isolated communities. Keiser et al. applied a mixed method, observational design to evaluate teamwork and communication following virtual/web-based deliberate practice and face-to-face simulation-based education of health service workers (39). Student evaluations were generally favorable, and the opportunity for multidisciplinary interaction was appreciated. In another program, Heginbotham et al. described an educational model using an online and in-person approach aimed at training parents, faculty staff and learners to ensure that children with special healthcare needs were receiving adequate home care (36). In the same vein, Miller et al. presented a descriptive case study detailing how virtual collaboratives were used to increase patient safety practices and quality of care, and to improve interprofessional collaboration in homecare settings (41). Characterized by few standardized routines and procedures, this part of the Canadian healthcare sector experienced a large proportion of patients reporting adverse and indecent treatment during their homecare. Most of these events were attributed to healthcare professionals’ failure to prioritize time and assignments, as well as insufficient information and training. The introduction of virtual collaboratives contributed to closing these gaps and raising awareness about safety practices in homecare (41).

### Innovation and improved interdisciplinary coordination

Several studies explored the increasingly complex nature of healthcare services, characterized by the need for interdisciplinary coordination and collaboration (30, 33, 35, 38, 40, 43, 45). In their study of intra-hospital care transitions, Wooldridge et al. applied process modeling and simulation inspired by human factors engineering methods to analyze roles and interdependencies in trauma care (33). To ensure quality care in complex healthcare systems, they proposed to strengthen clinical decision support at the individual level, to prioritize non-technical skills at the team level, and to enhance organizational awareness through process modeling and simulation. The study by Lama et al., further details the complexity and interdependence of highly specialized healthcare processes, by mapping and comparing diagnostic errors between clinicians and radiologists (40). Since radiological images are distributed and interpreted via electronic systems, radiologists and clinicians are seldom co-located. Lama et al., notes that an increasingly fast-paced, productivity-driven and fragmented healthcare system, presents systemic barriers to communication across professional and cultural barriers, which could pose an increased risk of misconceptions and adverse events (40).

The COVID-19 pandemic inspired a surge in innovative technology-driven approaches to the training, supervision, and transformation of healthcare services across geographically distributed teams (35, 38, 43, 45). The study by Anderson et al. discuss important preconditions that should be considered when providing radiation therapy across campuses and hospitals during the pandemic (45). They provided examples of critical risk-mitigating strategies that need to be addressed, and how workforce planning and communication are important for both patient and staff safety. To achieve this, the extended use of information-communication-technology becomes crucial. The study by Dhala et al. provides a timely example of how extended use of information-communication-technology becomes instrumental to implement and evaluate a program in support of virtual intensive care during COVID19 (35). In this program, virtual platforms were implemented to support remote monitoring and treatment of intensive-care patients in community hospitals. This virtual collaborative ecosystem contributed to increased patient safety and staff development.

Mental health services were significantly affected by COVID-19, and distancing requirements presented major obstacles to outpatient psychotherapy services. In their case study, Sasangohar et al. outlined how an outpatient mental health service decided to implement a 100% digital service, at the beginning of the pandemic (43). They described how logistical and technological issues, communication barriers and interpersonal relations, emerged as barriers to the therapeutic process and how these issues were addressed. Husain et al. provided a case study and evaluation of a web-based, asynchronous clinical communication system that was implemented to support team-based care (38). This web-based system (‘the Loop’) faced several structural and implemental challenges, from system integration to organizational and economic disincentives, which discouraged individual application of the system. To overcome communication barriers and to comply with infection control measures during COVID-19, virtual communication and live-streaming of ward rounds using wearable technology, were introduced into bedside intensive-care rounds. While this maintained social distancing and patient care, it also made it possible to provide remote education to medical students (24, 27). Participants reported that, even though technological solutions allowed for both audio and visual input during the ongoing case-discussions by the patients’ bedsides, these hybrid-rounds still were characterized by noise from the physical environment. Supervising doctors also were not able to physically assist the doctors in training, who in turn had negative effects on learning outcomes (29). Despite such barriers and technical shortcomings, the authors maintain that the hybrid-rounds method has potential to overcome its disadvantages, and thus may serve its purpose in situations where co-located teamwork is impractical or poses a health risk to patients and staff.

First-responders from the prehospital services must be prepared to perform lifesaving procedures in emergency situations that are, by nature, complex and challenging. Hughes et al. examined the effects of telemedical support on teamwork and cognitions in a simulated mass casualty event (37). Their study was not conclusively in favor of telemedical support under such circumstances, and more research is called for. Another study examined the efficacy of performing prehospital emergency anesthesia, including rapid sequence intubation, in a simulated aircraft on the ground, when wearing personal protective equipment (30). Despite the hassles associated with personal protective equipment, it had no significant impact on the time to successful completion of endotracheal intubation in this simulated setting, indicating significant patient benefits in terms of prehospital time savings and patient safety.

### Team processes and coordinating mechanisms in pre-hospital services

Three of the empirical studies of teamwork and team processes in this review, were performed by in-depth analysis of healthcare databases. Akşin et al. used data from the London Ambulance Service to investigate the impact of prior partner exposure on scene time, and patient handover at the hospital (34). For the less standardized patient pick-up process, greater partner exposure directly improved performance. For the more standardized patient handover process, the beneficial effect of partner exposure was triggered beyond a threshold of sufficient individual experience. In addition, the beneficial performance impact from prior partner exposure was amplified during high workload periods (34). This study provides empirical evidence supporting how shared mental models may contribute to patient safety in fluid teams, as the ambulance workers rotate and collaborate across different work schedules. This indicates that individual factors, such as trust and shared mental models, become increasingly important in high-intensity situations. The study by Johnsen, et al. utilized data from operators in emergency medical communication centers and first-line ambulance personnel to investigate the proposed shared mental model approach to teamwork (11, 19). A total of 240 participants from the ambulance service in a Norwegian city were used to study team effectiveness in 80 critical care missions. Path analyses showed that shared mental models were positively associated with team effectiveness, and negatively related to mission complexity. The coordinating mechanisms of shared mental models and closed-loop communication were positively related to outcome and team processes. In another study by Johnsen, et al., voice recordings from real-life ambulance missions were investigated for frequencies of coordinating mechanisms and team competencies based on differences in team behavior, between low and high-complexity missions (31). The results indicated lower frequencies of team competencies and coordinating mechanisms in high-complexity missions, than in low-complexity missions. The authors suggest that a lack of visual input from a team member during team interaction, could lead to team process loss and a team breakdown into sub-units, in high-stress situations (31).

## Discussion

Taken together, the 19 studies in this scoping review represent a diversity of research designs and methodological approaches to studying distributed team processes in the healthcare. A notable finding is the abundance of descriptive case studies or cross-sectional studies, while more rigorous longitudinal or randomized control trial designs, are absent. Several studies that focused on how virtual training sessions can contribute to inform healthcare providers in remote regions (42), or enhance interprofessional collaboration (31, 34, 36, 44, 46), could be followed up by experimental or longitudinal studies. With notable exceptions (19, 31), the majority of studies emphasized individual training outcomes, and were less focused on a conceptual or theory driven approach to team processes and outcomes, such as ‘The big five of teamwork’ (11).

A substantial number of studies were performed in North America (53%), followed by Europe (32%) and Australia (11%), with only one study (5%) from Asia, and no studies from Africa or South America. Furthermore, our results indicate that the COVID-19 pandemic spurred an increase in research on distributed team processes. Although our first search had identified 85 potential studies over a 10-year period, the supplementary search identified 88 additional studies over a two-year period. The COVID-19 pandemic clearly inspired a surge of research in this area, and most studies originated in North America and Europe. Several studies examined innovative approaches to the training and education of distributed healthcare providers, in which technological solutions were introduced to improve communication, coordination, and shared mental models in distributed healthcare settings. Among several benefits of distributed healthcare teams are more cost-effective, safe, and eco-friendly interactions when less time and resources are spent on travel and physical meetings (42). Another advantage of distributed teamwork is the opportunity to be exposed to diversity and other ideas and methods and to include training, supervision, and transformational outcomes into the virtual context (35, 38, 43, 45). Not surprisingly, barriers in communication and technology caused difficulties in coordination and the maintenance of shared mental models, indicating that ‘The big five of teamwork’ represents a viable model that should be further explored in research on distributed teamwork (11). This assumption is supported by the small proportion of studies which used health services data to examine team processes and coordinating mechanisms in distributed healthcare settings and prehospital services. Taken together, several notable findings from this scoping review should be considered to enhance future research on distributed team processes in healthcare:

- Ineffective communication is widely recognized as an important barrier in virtual teams. A more consistent application of communication taxonomy (e.g., closed loop communication) would allow comparison between studies.
- Likewise, several studies identified coordination issues to present a significant barrier to distributed teamwork in healthcare. Again, a more detailed classification of coordination activities will contribute to advance future research (e.g., mutual performance monitoring and backup behavior).
- Several studies have explored the effects and feasibility of technological innovations to enhance education, diagnostics, or patient care in distributed healthcare settings. These studies are typically exploratory in nature, have no control group and have a relatively small sample size. To advance research on distributed healthcare comparative studies of different technologies would be valuable.
- From research on team effectiveness, the concept of shared mental models has emerged as a key aspect in distributed teamwork. A future line of research would be to examine how distributed teamwork influence shared mental models across healthcare specialists with different professional backgrounds.
- Another strand of research would be to study distributed team processes across cultural barriers and how technological solutions could bridge cultural and professional barriers and improve access to high quality healthcare in low- and middle-income countries.
- Finally, this review points to the shortage of experimental studies, as well as the need to assess long-term trajectories and consequences from distributed teamwork in the healthcare services.

## Strengths and limitations

This scoping review followed the framework of Arksey and O’Malley, the PRISMA flow diagram and clearly determined eligibility criteria (27). This allowed a systematic process; whereby methodological considerations were considered before proceeding to the next stage. Multiple researchers assessed the outcomes, and the same three search words and databases were used in both main searches. The results clearly indicate that it was useful to conduct a second search to capture relevant research from the COVID-19 pandemic. Although the scope and outcome of the search may have been widened with a different search strategy or less-constrictive combinations of operators, the current strategy yielded a broad selection of studies that contributed to inform our research questions. A notable shortcoming is that most of the studies were reported from Europe and North America. This clearly indicate a need for encouraging more research from low and middle-income countries, which often must be dependent on geographically distributed and scarce healthcare resources. Hopefully, this review could encourage additional studies that explore barriers and benefits to distributed healthcare services in low-and middle-income countries. Another shortcoming is the absence of randomized controlled and longitudinal studies which could have contributed to causal inferences or identified long-term outcomes. However, we believe that this our review provides a preliminary assessment of the potential size and scope of the available research on common barriers and innovative applications of technology in support of team processes. It should be noted that while there are barriers in distributed team processes, there are also real benefits. In healthcare as well as in science and industry, decentralized, asynchronous teams accomplish extremely difficult tasks across continents and time zones. Thus, a better understanding of coordinating mechanisms and efficiency of geographically dispersed teams would benefit healthcare services and society at large.

## Author contributions

JE: Conceptualization, Data curation, Project administration, Resources, Supervision, Writing – original draft, Writing – review & editing. GB: Conceptualization, Funding acquisition, Project administration, Supervision, Writing – review & editing. JJ: Data curation, Investigation, Validation, Writing – original draft, Writing – review & editing. RE: Conceptualization, Formal analysis, Writing – review & editing. BJ: Conceptualization, Formal analysis, Funding acquisition, Investigation, Supervision, Writing – review & editing.

## Footnotes

^1^https://www.rayyan.ai/
